# A Treatise on Pathological Chemistry, or Chemical Researches on the Solids and Liquids of the Human Body, in Their Relations to Physiology and Pathology

**Published:** 1844-04

**Authors:** 


ART. X.

1. *Traité de Chimie Pathologique, ou Recherches Chimiques, sur les Solides et les Liquides du Corps Humain, dans leur rapports avec la Physiologie et la Pathologie.* Par S. D. L'HERITIER.—*Paris*, 1842. 8vo, pp. 744.

*A Treatise on Pathological Chemistry, or Chemical Researches on the Solids and Liquids of the Human Body, in their relations to Physiology and Pathology.* By S. D. L'HERITIER.—*Paris*, 1842.

2. *Lehrbuch der Physiologischen Chemie.* Von Dr. C. G. LEHMANN. 1ste Band.—*Leipzig*, 1842. 8vo, pp. 380.

*A Treatise on Physiological Chemistry.* By Dr. C. G. LEHMANN. Vol. I.—*Leipsic*, 1842.

3. *Anleitung zum Gebrauch des Mikroskopes zur Zoochemischen Analyse, und zur Mikroskopisch-chemischen Untersuchung uberhaupt.* Von Dr. JULIUS VOGEL.—*Leipzig*, 1841. 8vo, pp. 600.

*Introduction to the use of the Microscope in the Chemical Analysis of Animal Matters, and in Microscopico-chemical inquiries in general.* By Dr. JULIUS VOGEL.—*Leipsic*, 1841.

4. *Chemistry of Animal Bodies.* By THOMAS THOMSON, M.D.—*Edinb.* 1843. 8vo, pp. 702.

THE impulse given of late years to the prosecution of animal chemistry by the labours of Berzelius, followed in our own country by the accurate and successful researches of Prout, and in Germany by those of Tiedemann and Gmelin, and more recently by Liebig, among many other eminent names, is amply attested by the succession of works continually issuing from the press on this subject, both in our own and in other countries; and the importance of a careful application of the laws of inorganic chemistry to the study of the more delicate products of organized beings is at length claiming both from chemists and from medical practitioners its due share of attention.

It is hardly necessary, with the admirable practical treatise of Dr. Prout before our eyes, to insist upon the great value of chemical science, carefully and judiciously applied to the prophylaxis and management of disease. Indeed the danger now lies, as is frequently the case where a strong impulse in one direction has been given, in our running into the extreme of attributing more to chemical agency than justly lies within its reach, falling again into the errors of Van Helmont and his contemporaries, and vainly striving to cure all diseases by remedies, if any such there be, whose action is of a strictly chemical nature.*

* When we find an eminent professor attempting to explain the narcotic effects of morphia by its chemical composition, and referring to the same source to account for the febrifuge action of quinine, before we can at all conjecture why oxalate of potash should prove a deadly poison whilst the bicarbonate is comparatively innocuous—two substances which differ from each other only in the fact that the latter contains one equivalent more oxygen than the former—when we find the same chemist explaining the action of tea and coffee by a distant similarity in composition between caffein and taurin, an azotised crystalline principle extracted from the bile, not of man only who takes these articles, but also of herbivorous animals, who do not partake of them; it is pretty evident we are in danger of following the delusive glimmer of an ignis fatuus, in place of the steady light of unchanging truth.

A judicious and discriminating application of well-ascertained chemical facts to the employment of remedies in disease, must evidently prove a most valuable aid in the improvement of our methods of treatment; but we cannot be too cautious in sifting those facts, nor too wary in trying experiments founded upon hypotheses, recommended it may be by novelty, simplicity, and high authority, but which if erroneous might compromise some valuable life. Undue discredit would thus be brought upon science when the fault ought rather to be referred to the unskilful manner in which its principles were applied.

Fruitful as is the field thus opened to us, it can only produce its fruits by the proper cultivation of the soil ; and in this, as in all other cases, a certain amount of preliminary knowledge is required before those principles can yield the harvest which under right regulation they may be made to afford. *Physiological* chemistry requires among other things, a thorough acquaintance with animal chemistry in general, in which term we include *comparative animal chemistry*, i.e. the chemistry of different classes of animals, which is no less important to the chemical physiologist than comparative anatomy is essential to the student of microscopic anatomy. Lehmann's observations on this point are so judicious that we cannot avoid quoting them :

"Animal chemistry, (Zoochemie,) that is to say the knowledge of the chemical constituents of the animal body, bears the same relation to physiological chemistry that anatomy does to physiology; without anatomy we cannot conceive the existence of physiology ; and equally impossible is a physiological without an animal chemistry. But indispensable as animal chemistry is to the study of chemical processes in the animal body, yet it does not of itself constitute physiological chemistry. It is in itself not sufficient to the necessity, and even of but little service to the physiologist and physician, for it is deficient in its direct relations to physiological and pathological processes. Physiological chemistry first acquires value to the physiologist and physician, when each single chemical substance capable of isolation is considered in its relation to the entire organism, and in its influence on the vital functions, and when each chemical process is better understood in its connexion with the dynamics of the organism." (Pref. p. vii.)

It is equally evident that a sound physiological chemistry must prove the only solid foundation of a rational pathological chemistry, and the latter will of course shed a reflected light upon many unexplained points in the physiological part of the subject. Bearing these facts in mind, we shall be better enabled rightly to estimate the value of the works before us which (particularly the first three) profess to furnish us with facts and observations, more or less extensive, upon the different points connected with the physiology and pathology of the human frame; by thus affording us data whereby theories may be tested, they will prove of essential service to the practical physician.

M. L'Heritier divides his work into eleven chapters. The first, occupying more than a third of the volume, includes a lengthened consideration of the blood and its allied fluids, chyle and lymph. In the next he reviews the chemistry of digestion, and in the third passes to the urinary function in its healthy and diseased conditions. These subjects together occupy three fourths of the work. The remaining chapters are in succession devoted to the adipose, serous, and mucous tissues with their secretions; to the nervous system, external senses, products of the genital organs, and locomotive apparatus ; and the book concludes with a kind of appendix for such morbid products as do not conveniently find their place in the preceding classification.

Dr. Lehmann's work is upon a totally different plan, including pathological points only incidentally as they bear on physiological considerations. After a preliminary sketch of the chemical properties of organized matter, and the principal changes to which it is subject in the processes of fermentation, putrefaction, and decay, he gives a detailed account of the various ingredients, first of an inorganic, and then of an organic nature, which enter into the composition of the animal frame. The organic matters he divides into those essential to the constitution of the body, and those produced in the secretions or excretions. Each substance is considered successively : 1st, in its chemical relations, including its properties, composition, and mode of preparation ; and 2dly, in its physiological relations, under which are described the parts of the body in which it occurs, its uses, and its origin. The utility of thus clearly defining the different parts of the subject will be at once appreciated by those who may have occasion to employ this work as a book of reference.

The treatise of Dr. Vogel is again quite different from either of the preceding, and relates, as its title purports, principally to the employment of the microscope in chemical inquiries. The first fourth of the book is occupied with a description of microscopes of various kinds, the theory of their action and the mechanical management which each requires. Then follow directions for the performance of the simplest chemical operations, and descriptions of the requisite apparatus ; after which we have a detailed account of the proximate elements which occur in Zoochemical inquiries, the parts of the body in which they occur, physical properties, chemical relations, and analytical methods of determining the quantity in which they may be present.

The inorganic constituents are next briefly described, and directions given for preparing the various necessary reagents. General directions for organic analysis follow. The remaining third of the work is devoted to the application of the microscope to chemical investigations illustrated by examples of various kinds, with directions for preparing and preserving specimens for microscopic examination.

The work of Dr. Thomson is an elaborate compilation from the more recent memoirs on animal chemistry, reducing the subjects of them into a systematic form. It is arranged into three divisions : The first embraces the animal principles, and these are treated without much reference to their origin, under the heads of animal Acids, Bases, Oxides, Colouring matters, and Amides, or chief azotised constituents of the body. The second division of the work includes the solid parts of animals, the liquid parts, and morbid concretions. The third gives a brief review of the principal chemical functions of animals ; and an Appendix, contains an account of the methods of ultimate analysis now in use.

As the treatise of L'Heritier is that which bears most directly upon pathology, and therefore presents the most interest to the medical reader, we shall examine it somewhat in detail.

Commencing with the fluids concerned in sanguification, the author treats first of the *chyle.* Owing to the difficulty of obtaining it for examination even in a healthy state, however desirable such information would be, but little that is new has been done in relation to the analysis of this fluid. If our knowledge of it in a healthy condition is scanty, we are in still greater ignorance of its changes under the influence of disease. Thus much, however, is certain, that the chyle varies with the nature of the food taken, though the precise conditions under which it varies, and the changes produced have not been satisfactorily made out. Its composition closely resembles that of the blood; it is, however, considerably more dilute, and usually contains a much larger proportion of oily matter. Fibrin, albumen, a trace of the colouring matter of the blood, fatty matters *sui generis*, certain ill defined extractive matters, with water and the usual saline constituents of animal fluids, are its chief ingredients. The microscope discovers two distinct kinds of globules, one of which, varying in size and of high refractive power, is formed by fat held in suspension; the others closely resemble the colourless corpuscles of the blood. The amount of fibrin, (recognized by the tendency to spontaneous coagulation, and by the firmness of the coagulum,) increases, as the chyle passes from the walls of the intestinal canal to the thoracic duct; whilst that of oily matter diminishes.

With regard to that closely analogous fluid *lymph*, our knowledge is scarcely more satisfactory or accurate than that which relates to the qualities and composition of chyle; and L'Heritier adds but little to that scanty store. Like the chyle, its composition varies with the part of the system from which it is taken; the nearer it arrives to its point of admixture with the mass of circulating fluid, and the greater the time that has elapsed since taking food, the more concentrated it becomes It too contains globules, but they are smaller and rounder than those of the chyle. As a large part of the lymphatic system is less deeply seated than the chyliferous, its morbid conditions are more readily appreciated; but our information on this subject remains most vague and unsatisfactory. Dr. Rees has, by a comparative analysis of chyle and lymph in the same animal, (a young ass,) shown that the same animal matters are present in both, but that the chyle contains thrice the proportion of azotised matters furnished by lymph, with a much greater proportion of fat.

Next follows a long and diffuse chapter on the *blood.* We need not dwell upon the question of the vitality of this fluid which is here discussed. The cause of its coagulation is considered at some length, but little is added to our knowledge on the subject: The change is evidently the result of a vital, not a chemical act: chemical agents, therefore, when they destroy vitality, prevent coagulation. The author confirms the previous observation that the feebler the vitality of the animal which furnishes it, the more rapidly, *cæteris paribus,* but less firmly does the blood coagulate.

We pass next to the chemical constituents of the blood. Among the well-ascertained ingredients of this complicated fluid, the following may be enumerated as met with in its healthy state: Albumen; fibrin; red colouring matter; certain fats containing phosphorus; an azotised fat termed serolin; a minute portion of cholesterin; a volatile oily acid varying with the species of the animal, to which the peculiar odour of blood is due; besides a small quantity of oleie and margaric acids in the form of soap; chlorides, sulphates, lactates,* phosphates and carbonates of soda, potash, lime and magnesia; certain ill-defined, so-called extractive matters; besides free carbonic acid, the presence of which in venous blood, notwithstanding the denial of that fact by many high authorities, is clearly demonstrated by the experiments of Stevens and Magnus. No urea has hitherto been found in healthy blood. Many other substances are mentioned as having been found, but their occurrence in general is more than problematical.

M. L'Heritier cautiously abstains from adopting the opinion that fibrin and albumen are identical, and adduces their physical properties as well as chemical ones, as vouchers for his accuracy. Indeed the solubility of venous fibrin in an alkaline solution of nitre, though a striking experiment, does not amount to much in the way of proof, for it certainly is not equivalent, as some have supposed, to a conversion of fibrin into albumen, though in many of its reactions this liquid resembles a solution of albumen.

"It is indeed," remarks he, "of little consequence whether we regard fibrin and albumen as distinct and separate principles, or simply as modifications of the same principle. There is no doubt their offices in the economy are perfectly distinct, and it is probable the substances themselves are so likewise." (p. 74.)

The analytical researches of Dumas on the composition of these bodies, published since M. L'Heritier's treatise, tend to strengthen this opinion; as by numerous careful experiments, this distinguished philosopher found albumen invariably to contain more carbon and less nitrogen than fibrin. In one remarkable particular, fibrin, as Scherer and Mulder have shown, differs from albumen, viz., in its superior tendency to absorb oxygen. This absorption occurs rapidly when fibrin, whether in solution or in the solid state, is exposed to the air, whilst with albumen the alteration for a long time is scarcely perceptible. Doubtless this difference is intimately connected with the different share each of these principles takes in the processes of respiration, and the production of animal heat.

M. L'Heritier then proceeds with a detailed description of the physical and chemical properties of the more important constituents of the blood, which, as being recently noticed in this Journal, (review of Andral, in Number XXXIII,) we need not here repeat.

With regard to the changes of colour that the blood undergoes during respiration, he is a strong partisan of the opinion that "the salts contained in the blood are the true cause of the passage of the colouring matter from black to red, and consequently of the scarlet colour of arterial blood," (p. 96,) adopting Dr. Stevens's views, without mentioning his name.

The manner in which the oxygen of the atmosphere penetrates the parietes of the blood-vessels of the lungs, and is thus admitted to act upon the fluid they contain, would be more correctly explained, as Professor Daniell has shown in his 'Introduction to the study of Chemical

* Dr. Enderlin has recently published a paper in Liebig's 'Annalen der Chemie u. Pharmacie,' bd. xlvi, s. 164, in which he states that after a careful examination of considerable quantities of blood from herbivorous animals, he was quite unable to detect even a trace of lactic acid or of its salts. If this observation be confirmed and extended to the human species, the theories respecting the important part played by these compounds in the economy crumble to the dust.

Philosophy,' by the variation in the force of adhesion between the membranes, the different gases and the fluids which permeate the tissues, a result which Dutrochet, without sufficient foundation, considers to be due to the action of new forces. The results he designates conveniently enough by the terms *endosmosis* and *exosmosis*, indicating the mutual change of places effected between gases or liquids through the medium of a porous diaphragm; a process of high importance in the economy of organized nature. Some, however, have attributed effects to it to which it certainly can lay no claim. Thus, Liebig supposes air may pass directly from the stomach into the lungs. After speaking of the air which is carried down by the saliva, he says, "no doubt a part of these gases may enter the venous circulation through the absorbent and lymphatic vessels, and thus reach the lungs where they are exhaled, but the presence of membranes does not offer the slightest obstacle to their passing directly into the cavity of the chest." In another place, speaking of carbonic acid developed in undue quantity in the stomach, he says "the carbonic acid which is disengaged, penetrates through the parietes of the stomach through the diaphragm, and through all the intervening membranes into the air-cells of the lungs, out of which it displaces the atmospherical air." Surely this is carrying a convenient theory a little too far, to make carbonic acid pour into the lungs out of the stomach, as rapidly as from a brewer's vat into the store-rooms beneath, despite the intervention of stomach, peritoneum, diaphragm, pleura and all! During the process of respiration, nitrogen as well as oxygen is absorbed, and probably this is one source of the nitrogen exhaled by the skin. The nitrogen appears to undergo no chemical change, but is merely mechanically taken into the system from its solubility. Ordinary experiments do not discover this absorption; for if the blood be, as it usually is, saturated with nitrogen, the amount absorbed will equal that which is evolved, but by making animals breathe in a mixture of oxygen and hydrogen, nitrogen is always found in the respired air, being displaced from the blood by hydrogen. Carbonic acid and nitrogen are not the only substances thrown off by the lungs. It is well known that alcohol and many volatile and odoriferous bodies are excreted unchanged from the bronchial membrane during respiration.

We pass next to the methods of analysing the blood. The first plan described is that employed by M. Lecanu; it has less pretension, and is fully as accurate as the tediously minute process employed by M. Denis, which is quoted at full length from that writer. The following method is recommended by L'Heritier as being less elaborate, and therefore more practicable:

"We begin by determining the proportion of fibrin by whipping up a given quantity of blood on its issue from the vein; this done, we dry over the water-bath a known weight of the same blood, and weigh the residue to determine the quantity of water. In conducting this operation, care must be taken to preclude the admixture of dust suspended in the air or of any other foreign matters, by means of a gauze cover. Having exhausted the residue by ether, and boiling alcohol to separate fatty matters, we proceed to incineration in order to determine the proportion of oxide of iron which the ashes contain. This quantity once known, it is easy to deduce from it the amount of the hematosin contained in the blood, since Lecanu has demonstrated that the proportion of iron in the same quantity of hematosin is constantly the same. The proportion of this latter being determined, we may imagine the weight of the globules to be proportional to this quantity. We might also try whether the serum and fibrin contained traces of iron, and deduct this from the general sum. If we wish to estimate the proportion of albumen contained in the serum, we must coagulate a given quantity of it by heat; wash, dry, and weigh the coagulum." (p. 113.)

We will not say that M. L'Heritier has never practised his own process, but we do not hesitate to assert that the method here recommended for ascertaining the amount of red particles is quite inadequate to the purpose, as, according to the analysis of Denis, 1000 parts of human blood contain only 0·195 parts of oxide of iron, a quantity so minute that the errors inseparable from experiment would deprive any calculation of the quantity of the globules founded upon it of any scientific or practical utility. Moreover, the facts which this method of analysis furnishes are so meager as to be almost valueless. For all practical purposes the plan followed by Lecanu with the slight modifications adopted by Andral and Gavarret, is by far the best that has hitherto been proposed. The quantities operated upon in Simon's process are too small, and larger cannot safely be used. Our author next relates some experiments to ascertain the variation in composition of the blood taken from different parts of the same animal. His results confirm those previously obtained by Thackrah and Schultz, detailed by Mr. Ancell in his elaborate Lectures on the Pathology of the Animal Fluids, published in the 'Lancet' for 1840, and which contain an excellent digest of most that is known on these subjects. In five comparative experiments, M. L'Heritier found that the blood of the jugular constantly presented a larger proportion of saline matter than that of the vena cava, the blood of the portal vein compared with that of the jugular, contained, in five experiments, a considerably larger proportion of serum, and coagulated more rapidly. From portal blood he obtained by analysis less fibrin and fewer red particles. According to Schultz, the same blood furnishes nearly double the usual amount of fat. By experiments quoted from Denis, placental blood contains more red globules and less water than ordinary venous blood. The author adds nothing satisfactory to our knowledge of the difference between venous and arterial blood ; the only fact ascertained with certainty appears to be that a larger proportion of carbonic acid exists in the former. The subject requires reinvestigation and careful elucidation by comparative experiments.

A good review follows of the processes by which animal heat is generated, though the facts and reasonings adduced are such as have been long familiar to chemists: he draws the conclusion that the "principal cause of calorification resides in the combinations effected between the elements of the blood and the oxygen absorbed during the respiratory act, or disengaged in the texture (trame), the matrix as it were of the tissues, as a consequence and by virtue of the molecular reactions which constitute nutrition." (p. 136.)

Among other subjects reviewed by Liebig in his work on 'Animal Physiology,' the generation of animal heat has been treated at considerable length, and the conclusion he arrives at is that the chemical actions going on within the body are the *sole* source of the heat evolved ; an opinion supported by Dumas and other eminent men*—but it is not to combination alone with free oxygen that Liebig limits the source of heat; it is not simply to the union of atmospheric oxygen with the constituents of the tissues. He maintains that the transfer of *oxygen in combination* from one substance to another, is an important aid: *e. g.* in the conversion of the amylaceous or saccharine principle into fat, which is effected by the abstraction of oxygen from the elements of which it is composed. The oxygen abstracted in this process he imagines to be transferred to other compounds in the economy and during this act of transfer to give out heat; so that the *deposition of fat*, as well as its *absorption*, is a source of heat; a convenient method of turning oxygen to account under exactly opposite circumstances. We usually find that if bodies in *combining* give out heat, they on *separating* from such compounds absorb it again. If during the transfer oxygen evolve heat on uniting with a second body, whence could it obtain that heat but by absorbing it at the moment of separation from the body with which it was previously in combination. Dumas even denies the production of fat by animals; and then, of course, Liebig's theory and the objections to it fall to the ground. This eminent chemist has, however, demonstrated clearly that Dumas in his zeal to prove that all the proximate elements of the animal body are derived from the vegetable kingdom, has fallen into error; and that the generation of fat from bodies which do not contain it, is usually effected by the assimilating process, though there are instances in which it may be otherwise. We shall only briefly notice that portion of M. L'Heritier's work which relates to the important subject of the variations which the different constituents of the blood undergo during disease, as in our last Number we particularly directed the attention of our readers to these points, in the review of M. Andral's work.

The *watery part* of the blood appears to diminish in full plethoric habits, in inflammatory diseases, and especially in patients suffering from Asiatic cholera. In anemia and in hemorrhagic affections, it is increased.

*Albumen*, except in Bright's disease, where it is diminished, although subject to great variation, does not appear to follow any general rule. For more extended information on this point, as well as for details of the circumstances which modify the proportion of *fibrin*, the reader is referred to the article already alluded to. We cannot, however, altogether omit the recent important researches of Mulder on the buffy coat of the blood. Bouchardat imagined he had extracted gelatin from the inflammatory crust, especially in cases of rheumatism, by long-

* Whatever theory it may be fashionable to adopt, in order to explain the generation of animal heat, facts must not be disregarded, merely because they militate against that theory. The reader is therefore reminded of Chossat's remarkable experiment, in which, after dividing the spinal cord in animals, the temperature underwent a considerable elevation, an observation the accuracy of which was confirmed by Sir B. Brodie on repeating the experiment, and still further illustrated by a case reported by this eminent surgeon, in which, after severe injury to the upper part of the spinal cord, he observed in articulo mortis, when diaphragmatic respiration had continued only three or four times per minute for a considerable period, that a thermometer placed on the inside of the thigh attained the almost unexampled temperature of 111$^{0}$ F., where, with a very much diminished supply of oxygen, the heat far exceeded the normal standard.

continued boiling. This Mulder has shown to be an error, and has demonstrated by careful analysis, that what Bouchardat mistook for gelatin was a product of the decomposition of protein, and which has an elementary composition totally different from that of gelatin. He has further shown that the crust of inflamed blood consists not entirely of pure fibrin, but principally of this soluble substance which he calls tritoxide of protein, composed of C. 40, H. 31, N. 5, O. 15 + aq., which may be extracted by boiling, and of another substance insoluble in water, having the composition of deutoxide of protein, C. 40, H. 31, N. 5, O. 14. The quantity of *fatty matter* also varies, but according to no general rule; Simon, however, finds from numerous careful analyses that in inflammatory affections the quantity is sensibly increased, and usually in proportion to the increase of fibrin. The *salts* of the blood are observed specially to diminish during an attack of Asiatic cholera, and in the later stages of severe fevers.

The *red globules* appear to be the ingredients most subject to variation; and on this point our author confirms the observations of Andral. He finds that they are generally increased in plethoric persons, and in febrile attacks, and diminished in acute inflammatory affections, in anemia and chlorosis, and in all cases where imperfect nutrition or frequent loss of blood either by the lancet or otherwise has occurred.

We shall close this part of our subject with a few remarks on the state of the blood in one or two particular diseases.

The account of the blood furnished by our author in *morbus Brightii*, is incomplete, and considerably behind the knowledge we possess on the subject, thanks to the labours of Christison, Babington, and others of our fellow-countrymen.

The excellent researches of Bouchardat have at length settled the long-disputed question as to whether sugar exists in the blood in *diabetes*. He has shown that the quantity is greatest an hour or two after taking food, and that it gradually diminishes by continual elimination through the kidneys, so that if the patient fast for several hours, it almost entirely disappears. Diabetic blood coagulates loosely, is watery, and contains a smaller proportion of fibrin and red globules.

In *Jaundice*, the serum usually becomes coloured by the colouring matter of the bile, but the essential constituents of the secretion have not been found in it.

"The blood of jaundiced patients differs from the blood of other individuals, principally by the disagreeable taste of the serosity, and by the saffron colour of the latter, which becomes canary yellow when diluted with water. In jaundice coinciding with some inflammatory affection of the liver, of the lungs, &c. &c., not only the serosity is very deeply coloured, but the coagulum also presents a buffy crust tinged with yellow." (p. 238.)

In *tuberculous and cancerous* affections, the blood presents what Burdach has termed the albuminous diathesis. "Assimilation is abundant but incomplete, and it is marked by vermilion-coloured viscous blood, deficient in fibrin." (p. 261.) It coagulates therefore feebly and loosely, though sometimes it presents the coat,—the globules and saline matter are diminished in quantity, and the water increased.

In a solitary instance of *gout*, our author found urea distinctly in the blood, and as the proportion of uric acid in the urine increased, that of the urea in the blood diminished.

The aqueous portion of the blood in *cholera* is greatly lessened, so that the quantity of solid matters is in some cases nearly doubled. The salts, and particularly the alkaline carbonates, are also much smaller in quantity; urea is almost universally present, and the fluid is black and has a tarry consistence.

Lecanu found that in *endocarditis* and *pericarditis*, the globules were diminished and watery portion increased,

Of the state of the blood in *syphilis*, little is known, as an opportunity is seldom afforded of examining it uncomplicated by the action of remedies. In secondary syphilis our author found a considerable diminution of the globules, and a remarkable irregularity in their form.

A peculiar *milky appearance* of the blood has now and then been observed due to the presence of an extraordinary quantity of fat in this fluid, which has been found to constitute from three to five per cent. of the serum. The serum of this blood is less dense than usual, and contains less albumen than the normal quantity.

On the effects of certain remedial and poisonous agents upon the blood, the information we possess is still less precise or extensive. Our author devotes a short section to this subject. He makes the following remarks upon poisoning with carbonic acid:

"The researches which I have published upon this species of asphyxia have conducted me to results entirely opposed to those which authors up to that period had described; thus, whilst all agree in saying that the venous blood of subjects asphyxiated by the vapour of charcoal is of a dark black, I have always found it rosy or cherry red. The small veins which ramify under the skin of the animals experimented upon (rabbits) presented themselves under the form of ramifications of a pale rose colour. I believe the contradiction which exists between my observations, and those of my predecessors is to be explained by the rapidity with which the asphyxia supervenes, and especially by the period at which the examination, post mortem, is performed. When it is deferred, the blood which at first was a bright rose colour, assumes a bluish tint. In animals which are opened immediately after death, this phenomenon is very remarkable: immediately the intestinal mass comes into contact with the air, the vessels which traverse it lose their bright rose tint, and become livid; the same occurs with the intestines themselves, and with the mesentery. From these remarks I have concluded that asphyxia from charcoal is not a *negative asphyxia*, as has been said, but truly an asphyxia with poisoning of the blood." (p. 278.)

It has been long well known that carbonic acid does not produce poisoning by negative asphyxia; animals allowed to breathe common air but confined in a bladder, so that the whole surface of the body except the head is exposed to an atmosphere of carbonic acid, quickly perish, with all the signs of asphyxia from the gas.

In a variety of cases of poisoning the blood is evidently materially altered in appearance, consistence, and smell, although, from the want of chemical analysis, our information is devoid of all scientific precision. Although oxalic and hydrocyanic acids have never been found in the blood by chemical tests, yet the poisoning of leeches applied to the body in these cases lends some probability to the opinion that they exist as such in that fluid.

We pass now to the second chapter, comprising "the products of secretion, which concur to digestion, the products of digestion, and the digestive tube, with its adjuncts."

*Saliva.* This fluid is, in health, generally slightly alkaline, though during dyspepsia, febrile and inflammatory affections, and in malignant diseases of the stomach, it generally assumes an acid character. Among its constituents M. L'Heritier enumerates water, salivary matter or ptyalin, ozmazome, [?] with lactate of soda, mucus, chloride of sodium, phosphate of lime, and silica. He considers as problematical the presence of sulphocyanic acid announced by Gmelin; we have had, however, frequent occasion to prove its presence; and the elaborate experiments of Dr. Wright, in his excellent examination of the saliva, have shown that the quantity of sulphocyanides is increased by the exhibition of free sulphur. We have but few chemical analyses of the saliva in disease, and those few relate principally to the proportion of water and solid matters. Healthy saliva contains on an average 98·65 water, 1·26 of organic matters, and 0·09 of fixed saline matters, according to M. L'Heritier. In mercurial salivation the mucus is increased, the salts and ptyalin remain the same. The composition of healthy saliva is, however, very variable, as the proportion of solid matters may be diminished one half without any deviation from health. In diabetes sugar is occasionally present in the saliva, and in Bright's disease urea has been detected in it. Lehmann (p. 286) states that in cases of diabetes he has found lactic acid, in its free state, in the saliva, and that he believes its acidity is a very constant symptom.

We find little that is new in relation to the *gastric juice*, excepting that our author disputes the important part performed by pepsin in the act of digestion, and erroneously attributes to all mucus mingled with acids a similar power. As is well known this fluid, when the stomach is empty, is small in quantity and nearly neutral; if the viscus be irritated by the presence of food or by any stimulus, it becomes acid. The *intestinal secretions* are briefly described in the following words:

"The part of the digestive tube described under the term intestines, is also lubricated by a liquid which contributes, like the gastric juice, to the digestion of the aliments taken. This juice, neutral when the viscera are empty, becomes acid and more abundant during digestion, especially in the jejunum. In the large intestine, on the contrary, except in the cæcum, it is feebly alkaline. The impossibility of procuring this liquid in a state of purity is the reason that we have no accurate analysis of it." (p. 315.)

The nature of the acid of the gastric juice has been the subject of much dispute. Dr. Prout found it to be principally hydrochloric acid, a result confirmed by Tiedemann and Gmelin, who found likewise a variable proportion of lactic acid, which is considered by Dr. Prout, however, as a morbid product.

*Pancreatic secretion.* On this subject, the experiments of Tiedemann and Gmelin are quoted, but nothing new is added.

*Bile.* The elaborate analysis of the bile by Gmelin is also detailed, but our author judiciously remarks that a great number of the substances obtained by this chemist are doubtless the result of operations performed in the laboratory, an opinion abundantly confirmed by the recent researches of Berzelius. According to this celebrated chemist, ox-bile consists of the following substances: 1, mainly of a peculiar resinoid substance termed by him *bilin*, which is the most characteristic constituent of the fluid, (a substance which Liebig considers a resinous acid in combination with soda,)

with a small portion of a resinous acid, the bili-fellinic; 2, mucus of the gall-bladder; 3, a peculiar colouring matter, identical in properties with chlorophyll, (the green colouring matter of the leaves of plants), and like it susceptible of three distinct varieties, of which the green and the brown are of frequent occurrence; 4, small quantities of fatty acids, (the stearic, margaric, and oleic;) 5, a minute portion of cholesterin not exceeding $\frac{1}{10,000}$; besides, 6, free soda and phosphates, chlorides, and sulphates of soda, potash, and lime. From the facility with which the bilin undergoes change, and from the complex nature of the secretion, notwithstanding the numerous researches of which it has been the subject, our knowledge of it even in the healthy state is very imperfect, and although liable to numerous and important changes in disease, our information respecting the chemical alterations it undergoes is limited and deficient. Of the uses of the bile we are almost as ignorant as of its chemical composition. Tiedemann and Gmelin have given the most probable explanation of one of its chief objects, viz., a means of separating the superfluous hydrogen and carbon from the system, that the lungs may not be over-burdened. In some of the lower animals, as the doris and tethys, the gall-duct opens close to the anal aperture, and we find in hot weather and in hot climates, where the respiratory function becomes more sluggish, that the liver shows greater activity, and, from the increased share of duty that falls upon it, is liable to enlargement and disease. Liebig, however, has started the hypothesis that the bile is principally subservient to respiration, and that the chief office of the liver is to generate a material for the support of animal heat. All the facts with which we are acquainted, the considerable proportion of bile in the excretions, (though this is denied boldly by Liebig,) the history of diseases of the liver, the anatomical peculiarities of the doris and tethys, &c. &c., militate so strongly against this view, that it probably would never have obtained credit but from the high authority whence it emanated. It is to be observed that bile is never found in healthy blood. Simon has shown that a very small quantity causes destruction of the blood-globules, and it is doubtful, even in disease, whether more than the colouring matter has ever been certainly proved to be present, except, perhaps, in those remarkable cases of jaundice, whose pathology was first elucidated by Dr. Alison, in which death rapidly supervenes from the accumulation of *all* the elements of the biliary excretion in the blood, owing to a complete suspension of the secreting process. Our knowledge of the morbid alterations of the bile is scanty and deficient in precision. Thus it varies greatly in *colour*, being green in the diarrhœa of infants and black in yellow fever (?) in *consistence*, being much less aqueous than usual in cholera; and in *quantity*. Some interesting remarks are given on the connexion between the skin, lungs, and liver, for which our readers are referred to the treatise itself; the chapter well merits perusal.

Next follows a section on the act of digestion. As, however, it is a mere recapitulation of facts already systematized in various treatises on physiology, it need not here detain us.

*Products of digestion.* The chyle having been already described, the feculent matters are first considered. These amount, on an average, to 6 oz. daily, three quarters of which are dissipated by desiccation; about thirty grains of the residue are biliary matter, according to the analysis of

Berzelius. They are estimated at from 1-11th to 1-20th of the food, solid and liquid together, or at 1-7th or 1-8th of the solids taken. Generally speaking, unless abounding in bile, they are neutral in their action on test paper. The composition of these excretions varies with almost every malady, and the appearance, odour, colour, and consistence, betoken important changes, with which every practitioner is familiar; dependent upon the superabundance or deficiency of the bile, the greater or less amount of the mucous secretion of the intestines, the nature of the ingesta, &c. &c. But little, for obvious reasons, has been effected in the way of chemical analysis. Simon has recently published a recapitulation of what has been done by different authors as well as by himself. He found the solid parts of the *meconium* to contain after desiccation as much as 16 per cent. of cholesterin in crystals, besides a large quantity of bili-fellinic acid. In a case of cholera, he found but little albumen in the stools, which contain 98 per cent. of water and 1·3 of inorganic salts. Mr. Ancell, on the authority of Dr. Ainslie, states the alvine evacuations in this disease to be strongly alkaline, white, fluid, flaky, and containing the salts of the blood. Other observers have found sugar in the fæces of patients suffering from diabetes mellitus, and it is doubtless for want of examination alone that numerous other important alterations have escaped notice.

We come next to the results of some interesting observations on the gases of the intestinal canal. These have been found to vary according as they are examined, higher or lower in the alimentary tube. Thus in the stomach in health three quarters consist of nitrogen, the remainder of oxygen, hydrogen, and carbonic acid; the principal part being evidently due to the air swallowed. In the intestines, hydrogen, sulphuretted hydrogen, carbonic acid, and nitrogen occur in varying proportions; part of these gases are derived from the decomposition of the food, and part, there is little doubt, from a process of actual secretion, as in many cases of hysterical flatulence. Some experiments of M. Chevillot, combined with those of the author, on the proportion of the different gases in morbid and other circumstances, are detailed at length. Carbonic acid appears to be most abundant in persons suffering from acute and pulmonic affections.

"Subjects who have sunk principally under phthisis yield generally little or no hydrogen, and that this gas is found especially in those subjects who have died of acute diseases or of affections of the digestive system. The nature of the food or medicines administered to the patients appears to us to have great influence upon the production of hydrogen, but the difficulty of appreciating it on account of the variety of food and of the medicines, causes it to be difficult to give an account of it." After detailing some experiments, he says, "Hence we may, perhaps, conclude, that in general the vegetable acids, spirituous or ethereal liquors oppose the development of hydrogen, or at least produce very little of it. ...... This gas is generally more abundant in the small intestines than in the stomach and large intestines." (p. 386.)

Next follows a short chapter, containing the results of analyses of the *liver* and *pancreas*. The analysis of the *glands* is a subject replete with interest, and well worthy of accurate investigation, as it bears directly upon the important process of secretion, and more particularly upon a theory which is gaining ground among physiologists, and which has been ably developed by Mr. Bowman, in his article on Mucous Membrane, in one of the late numbers of the 'Cyclopædia of Anatomy and Physiology.' Our information on the subject is, however, meager. In an analysis of a healthy liver, by Braconnot, we find 18·94 of parenchyma, 25·56 of soluble matter, and 55·5 of water; of the soluble matter 3·8 per cent. was fat, and 20·19 albumen; no biliary resin is mentioned. Vauquelin found in fatty degeneration of the liver 45 per cent. of semi-solid saponifiable fat, only 36 of water, and 19 of parenchyma.

In the analysis of the pancreas and salivary glands nothing worthy of comment is found.

*Urine.* The third chapter is devoted to the Urine and Kidneys.

As, however, the attention of our readers has been very recently called to this subject in the analysis of Becquerel's valuable treatise on the 'Semeiology of the Urine,' of which our author has made copious extracts and abundant use, our observations upon this head will be principally supplementary to those already referred to.

In consequence of the variation, in composition of the urine at different times according to the longer or shorter period that has elapsed since taking food, our author recommends the adoption of Chossat's suggestion, employed so successfully by Lecanu and Becquerel, of collecting the urine of the whole twenty-four hours, and experimenting upon the mingled mass. As Becquerel's tables for the density and solid contents of the urine are quoted at length, it is necessary for us to caution the reader against their too ready adoption, as though Becquerel's tables are accurate for saline solutions, the one furnished by Dr. Henry is much nearer the truth for organic matters, especially in cases of diabetes, to which it professes alone to refer. The mean density of the urine is variously stated by different writers. The following precautions are quoted from Becquerel:

"To obtain approximatively the mean density of the urine, it is necessary always at the same time to give the quantity of urine passed, and thus placing these two elements together, that is to say the density and the quantity of the water, we have averages sufficiently exact, and we give an explanation of the differences found by authors in the appreciation of the density of urine in a state of health." (p. 411.)

The mean quantity of water and solids found by L'Heritier scarcely varies from that of Becquerel; for in twenty analyses taken indiscriminately from either sex, he found the solids to range between 506 and 618 grains. It is worthy of notice that when the quantity of water is increased, as by copious draughts of pure water, the proportion of solid matters, even in health, is increased, though by no means in a ratio corresponding with that of the water evacuated. These solids of the urine consist of

"Urea, uric or lithic acid, lactic acid, acetic and butyric acid, urobenzoic acid, lactate of ammonia, ozmazome, extractive matter soluble in water only, vesical mucus, sulphate of potash, sulphate of soda, phosphate of soda, phosphate of ammonia, chloride of sodium, muriate of ammonia, phosphates of lime and magnesia, colouring oil, a little fatty matter and debris of epithelium. The urates are excluded from the constituents of the urine in opposition to the opinion of Prout; it is however probable that they exist in this liquid, but in very small quantity." (p. 423.)

Upon the changes the urine undergoes in disease, we have little to add to the numerous and careful investigations of M. A. Becquerel.

According to this author, the *urea* in health may vary in the twenty-four hours from 222 to 278 grains, on the average, which, however, is greatly influenced by the diet of the patient. M. Lehmann has given us some experiments upon his own person bearing directly upon this point. He, however, always found a larger quantity of urea in his urine than that indicated by almost all other chemists. After taking nothing but hard boiled eggs for three successive days, he found the quantity of urea he excreted daily increased from 505 grains to 821·5 grains; during a vegetable diet it diminished proportionately.

After some directions on the analysis of the urine, a detailed and rather diffuse account is given of the various substances not normally present in this secretion, which occasionally find their way into it, embracing the methods of chemically detecting their presence, and their value to us in guiding or assisting our diagnosis.

*Blood* often occurs in the urine, both in local affections, such as calculus, and in constitutional diseases, as scurvy and fever. Its presence is recognized by the colour and the appearance of globules when examined by the microscope. Lecanu's process for chemically detecting its occurrence, is given as follows:

"The urine, previously saturated with a little nitric acid, if it be ammoniacal, is raised to ebullition; the coagulum formed is collected on a filter, and treated on the filter itself, if it cannot be detached from it, with alcohol acidulated with sulphuric acid; it becomes decolorized whilst the liquor takes a brown tint which passes to a lively red by a slight excess of ammonia. On evaporating the alcoholic solution, the colouring principle is collected on the surface of the liquid under the form of a black matter of resinoid aspect, soluble in acetic ether and in ammoniated alcohol, to which it communicates a red colour, and which gives with muriatic acid a yellow solution precipitated blue by the addition of ferrocyanide of potassium. The red matter of the blood is thus completely characterized by its peculiar properties and by the reaction of the iron which it contains." (p. 469.)

*Albumen* may occur in urine which is acid, alkaline, or neutral, and when its presence continues for any length of time it always indicates serious disease, most generally congestion, inflammation, or granular disease of the kidneys. It may occur also after chronic affections of the heart and liver; and it has even been observed in chlorosis, anemia, and scurvy.

"This phenomenon coincides most commonly with the presence of pus, of blood, of semen, &c. in this liquid; with asthma, certain diseases of the heart and of the liver; with an acute febrile disease; with a non-febrile disease accompanied by great functional disorder; with a considerable number of dropsies; with various organic or functional alterations of the kidneys; and finally with a general alteration of the circulating fluid........... As to the presence of albumen in the urine, considered as an element of diagnosis, it is doubtless of great value; but we must at the same time pay attention to the other modifications which this liquid may have undergone. Thus in Bright's disease, the urine is albuminous, of small density, slightly charged with urea, uric acid, and salts; in some acute diseases, on the contrary, albumen shows itself in a transient way, but the specific gravity of the liquid is considerable, and there is an excess of uric acid and saline matters.

"Urine which is at the same time albuminous and sanguinolent, indicates for the most part hemorrhage from the urinary passages, fungus, cancer of the kidneys or calculous pyelitis. That which is at the same time loaded with albumen and with mucus, is almost always connected with chronic inflammation of the bladder, of the ureters, of the pelvis, or calices of the kidney: finally, purulent and albuminous urine constitutes a common symptom in chronic pyelitis, renal calculi, abscess of the prostate or of the neighbouring parts, &c." (p. 477.)

A peculiar *viscous urine* has been met with, of spermatic odour, which contained a substance resembling albumen, fibrin, and mucus, in its proportions, but which corresponded exactly with none of these substances. In *chylous urine* a gelatinous coagulum, of variable size and consistence, indicates the nature of the disease.

"Urine of this nature, it is said, for I have never observed it, may be recognized by its containing *globules*, (analogous to those of the blood, but smaller, and colourless or pink, soluble in acetic acid,) albumen, imperfect fibrin, and a certain quantity of fatty matter." (p. 479.)

An observation of Dr. Prout's that fibrin may occur in the urine in sufficient quantity for the liquid to form a solid mass, is disposed of in a very cavalier manner: "I am not aware that this *opinion* [*!*] has been confirmed by other experimenters, and I *doubt* if it ever is so."! (p. 478). A mere *doubt* of M. L'Heritier's against an *observation* of one of the most accurate philosophers and experimenters that ever lived,—savours something of presumption; and we may be permitted to *doubt* whether M. L'Heritier's experience be quite as extensive as that of Dr. Prout, especially as he admits that *he has never seen* a case of chylous urine. The occurrence of *milk*, or its characteristic principle, *casein*, has never been satisfactorily proved, though often asserted.

*Kejestein.* This term was given by M. Nauche to a film he observed on the morning urine of pregnant women, after allowing it to stand undisturbed twenty-four or forty-eight hours. He considers it an infallible test of the existence of pregnancy, but has not examined it minutely. Dr. Golding Bird has made some experiments upon the subject, but the most complete account of its properties is given by Lehmann at p. 252; part of his description we here quote:

"By observation of the urine of a woman from the first to the seventh month of pregnancy, I found the urine of a dirty yellow, and more disposed to froth than usual; it generally became turbid in from two to six hours; upon the morning urine, at the expiration of thirty-six or forty-eight hours, a grayish white film always appeared which, often in from two to three days, sank down upon the sediment formed from the commencement of the turbidity. I always succeeded in extracting by ether no inconsiderable quantity of a semi-solid fat, which when saponified by potash and decomposed by sulphuric acid, emitted a most decided odour of butyric acid." [The residue of the film insoluble in ether showed by experiments which it is unnecessary to detail, that it was a protein compound differing in properties from albumen.] "From the foregoing experiments," remarks M. Lehmann, "we may conclude with certainty, that Nauche's kejestein is no new peculiar substance, and nothing more than a mixture of butyraceous fat, phosphate of magnesia, and a protein compound resembling casein."

The *spermatic liquor* can only be satisfactorily demonstrated in urine by examining the deposit from the fluid by means of the microscope; the existence of the spermatozoa then shows its presence without doubt.

Urine which contains *pus* is always more or less opaline, and in the course of a few hours, at the latest, deposits a yellowish or whitish stratum, which by agitation is easily suspended in the liquid. Heat and nitric acid cause coagulation from the albumen pus always contains; alkalies convert the deposit into a ropy, gelatinous liquid. Purulent urine quickly becomes ammoniacal, and hence undergoes a change by which the pus is converted into a tenacious glairy mass resembling mucus. In fact, Dr. Babington, by agitating pus with a solution of common salt and muriate of ammonia, and allowing it to stand some time, then adding a little free soda, and passing carbonic acid gas through the liquid to saturate the alkali, obtained a tenacious glairy fluid, which coagulated with acetic acid, and which he was quite unable to distinguish from genuine mucus.

"Microscopic inspection permits us to discover in the deposits of purulent urine globules more voluminous than those of those of the blood, reunited into a mass one over the other, and so confused and nebulous, that it is impossible to distinguish them exactly except upon the borders of the mass. But we should have a very incomplete idea of these corpuscles if our examination terminated here. Generally we place under the lens a drop of the layer of urine immediately above the purulent deposit. Here we perceive globules separated or reunited in groups, of variable size and opacity, rounded, with borders more or less regular, having a whitish granular surface, dependent, according to M. Vigla, upon a certain number of small grayish grains contained in these globules. These little bodies have not always the regularity here assigned to them; the more alkaline the urine becomes the more misshapen they become, and the more does their circumference appear notched; some are even reduced to half, a third, or a fourth of their size, a circumstance which helps to give them very extraordinary outlines." (p. 487.)

Our author gives a singular case from his own practice of apparent absorption of pus from an abscess, and its evacuation from the bladder with the urine:

"A woman named Delage, enrolled among the number of the poor in my division, sent for me on the 18th of September last; she complained of acute pain caused by a tumour which had formed during the last three days below the scapula about an inch from its inferior angle. The examination of this tumour immediately convinced me that it was formed by a purulent collection. Indeed a small puncture was followed by an abundant flow of laudable pus; I closed the incision with adhesive plaster, and ordered emollient applications. Two days afterwards a fresh accumulation of pus had taken place in the sac, and as I was preparing again to empty it by puncture, the patient, aged 86 years, entreated me not to use the instrument upon her again. The great age of the woman induced me to pay attention to her request; and I consented to exchange my lancet for caustic potash. I caused a piece the size of a pea to be applied to the tumour; twelve hours after, the tumour had completely disappeared; not because it had been opened by the caustic, not that it had emptied itself by the small incision I first made, and which had closed; but truly by absorption. During the night, fever had occurred, *the urine showed itself turbid and sedimentous;* I analysed it, and *found a matter to which it was easy for me to attach all the characters of pus.*" (p. 489.)

The colouring matter of the *bile* is discovered in urine by the more or less intense yellow tinge it communicates to the fluid or to linen stained with it. Nitric acid which causes it to pass to green, or reddish brown if in excess, has been proposed as a test for it; it is not infallible, and is seldom necessary. If employed, it is best, as Berzelius proposes, to evaporate the urine to dryness, treat with alcohol, evaporate this tincture to dryness, and then add nitric acid.

*Sugar* is pathognomonic of diabetes mellitus; its presence is best detected by fermentation with yeast, and if a given weight of urine with yeast be inverted in a graduated jar over mercury, the quantity of sugar may be determined with considerable accuracy by estimating the quantity of gas evolved, taking the necessary precautions to ensure exactness.

A variety of substances given as medicines pass into the urine, and may be recognized in the secretions; among those well ascertained are iodide, cyanide, ferrocyanide and sulphocyanide of potassium, sulphates of potash, soda, and magnesia, tannin, indigo, and quinine—various colouring and odorous principles. Many medicines are acted on by the economy, and pass out by the urine, having undergone change in the transit. Thus benzoic acid is separated as hippuric acid, and many salts of the vegetable acids as the *tartrate* and *citrate* of potash, occasion the presence of *carbonate* of potash abundantly in the urine. It is doubtful whether iron, mercury, arsenic, and antimony do pass into the urine; it is most probable that they do not.

*Clouds* and *sediments* from the urine are classed by our author according as they are deposited from acid or alkaline urine.

"The clouds which are observed in *acid urine* at the moment of their emission may consist of mucus, pus, seminal fluid, blood, epidermic scales, urates and uric acid held in suspension in the midst of this liquid............. The *sediment* from acid urine may be composed of uric acid, urate of ammonia, phosphate of lime, oxalate of lime, chloride of sodium [?] cystic oxide and mucus, mingled, combined with a certain quantity of the colouring matter of the urine." (p. 510.)

Into their chemical and microscopic characters we need not enter, as they are already well known to the profession through the medium of Dr. Prout's invaluable treatise.

"*Alkaline urines* are generally turbid at the moment of their emission. This appearance is due to the insoluble salts they hold in suspension........... The sediments of alkaline urines are usually composed of mucus, of phosphate of lime, alone or mingled with ammoniaco-magnesian phosphate; in some rare cases urates and other earthy salts are found also." (p. 513.)

For the methods of distinguishing the various sediments, the reader is referred to the work itself. (pp. 513 bis, et seq )

It is not surprising that diseases of the kidneys should materially alter the composition of the urine; the characters it assumes vary greatly with the stage of the malady. In *nephritis* it generally takes the febrile type with great diminution of the quantity of water; occasionally blood makes its appearance. Chronic nephritis often furnishes an alkaline urine loaded with phosphates. The modifications the urine undergoes in morbus Brightii need not here detain us, as our author adds little new on these cases; urea and the peculiar organic matters are diminished, and albumen and sometimes blood make their appearance. In *cystitis* mucus, in varying quantity, is found, so that the urine quickly putrefies; in chronic cases the secretion is usually alkaline and muco-purulent, frequently containing earthy phosphates. In acute *cerebro-spinal* affections the urine is generally febrile; when they assume a chronic form it not unfrequently becomes alkaline. In *diabetes* the quantity of sugar varies much with the nature of the food taken; a little blood is occasionally present, and frequently an imperfect form of albumen; and urea, though in some cases diminished, may always, when due care is taken, be detected. According to Bouchardat, quoted by our author, the proportion of sugar and urgency of the thirst varies directly with the quantity of amylaceous matters taken; an assertion which, however, requires some limitation, as Dr. Gregor has shown that the stomach in this disease possesses the power of converting even animal matter into sugar. This disease, therefore, is in great measure one of primary assimilation.

Next follows a chapter on the Cellular and adipose Tissues. M. L'Heritier has analysed the fluid effused in various forms of dropsy; he found it generally alkaline, with a specific gravity, varying between 1010 and 1017, always albuminous, and containing from eight to twelve per cent. of this matter.

*Fat.* The following remarks on the deposition of fat are worthy of notice, though not true in their fullest extent:

"Without speaking of the relations which regulate the biliary secretion and the development of fat, I ought to recall here the influence which respiration, that contributes so much to the elaboration of the blood, exercises upon the qualities of the material products of the organism. Thus when it becomes less lively, the blood assumes a more venous character, and the production of fat becomes more active. The seals and cetacea, animals whose blood is venous in the highest degree, are also abundantly furnished with fatty matter. In a word, every thing which augments venosity, especially a lengthened stay in a moist atmosphere, seldom renewed, favours the production of fat, and it is for this reason that aquatic animals are in better condition than those that live in the air." (p. 573.)

The effusions into the serous membranes greatly resemble those into the cellular tissue, being for the most part alkaline, and containing variable proportions of albumen; the fluid from the pleura, pericardium, and peritoneum is more concentrated than that from the other serous membranes. The synovia, except in consistence, does not differ from serous effusions in general in any important particular.

The *mucous membranes,* though their secretions offer modifications of colour, consistence, and appearance, which are familiar to the practical physician, and form valuable guides in prognosis and treatment, have as yet been but superficially examined in their chemical relations. The expectoration from the lungs is especially worthy of careful examination, as it cannot fail, with due attention, to afford valuable aid in diagnosis. The results of a microscopic examination of different forms of expectoration are detailed by Vogel. (pp. 421-4.) The spurious melanosis, or "black spit," to which miners are subject, it is fully proved, depends upon carbonaceous particles introduced from without during the act of respiration. The microscopic characters of mucus in general vary with the nature of the part from which it is taken. Epithelium scales are generally found in it abundantly, besides numerous spherical transparent granular masses which have received the name of mucus-globules.

Still less satisfactory is the state of our knowledge respecting the variations in composition of the *nervous centres* in health and in disease. Some comparative analyses of the brains of idiots and of persons in health have been made by our author, and although few in number, it is worthy of remark that the quantity of phosphorus which he found in healthy brains to be about 1·8 per cent., he observed in the idiot to be only 0·85. A similar diminution has been observed by Couerbe. It is stated, on the authority of John, that the gray matter of the brain is altogether devoid of phosphorus.

The next chapter is devoted to the organs of the External Senses.

*Sweat.* The secretion from the skin is for the most part acid, and in its healthy condition contains from 0·5 to 1·4 per cent. of solid matters, composed of free lactic acid and lactate of ammonia, animal extractive matters, chlorides of potassium and sodium, phosphate of lime, besides free carbonic acid and nitrogen gases, which are volatilized along with the water. In disease its qualities vary greatly; thus in lying-in women, in children troubled with worms, and in women before menstruating, the quantity of free acid increases remarkably. In jaundice it is often coloured yellow from the colouring matter of the bile; while in persons suffering from gout, lactic, phosphoric, and uric acids have been found. The secretions from the diseased surface in various cutaneous affections have been superficially examined, but yield no striking results; and the same may be said of the other organs of the external senses.

Next follow the Products of the Organs of Generation.

*Semen.* It is sometimes necessary in medico-legal investigations to ascertain whether spots on linen be due to the spermatic fluid or not. If due to the seminal liquid the linen is stiff, and when moistened, the peculiar odour of the secretion becomes perceptible; on holding the linen to the fire the stains become distinctly yellow. Macerated in a small quantity of water, and then the fluid examined by the microscope, the spermatozoa may generally be recognized.

*Milk.* This fluid, the most important aliment in a physiological point of view, inasmuch as it is the only one which contains everything necessary to the support of life, consists of the same substances in all the mammalia, although the proportions in which they occur vary considerably. In the human female the azotised matter, casein, constitutes from 1 to 2 per cent.; butter, from 3 to 8 per cent.; sugar of milk, from 7 to 8 per cent. It is always alkaline, and among the salts, the quantity of which is from 2 to 2·5 per cent., are free soda, chlorides of sodium and potassium, and phosphates of lime, magnesia, and iron, the latter in very minute quantity. Late researches appear to have shown that the solubility of the casein depends on the presence of alkali in combination with it. The composition of milk varies with the period that has elapsed since parturition. That first furnished, known as colostrum, is denser than that which flows subsequently; it becomes gelatinous on the application of heat. According to the researches of Simon on human milk, it contains twice the usual quantity of fat, and half as much again of sugar, besides a larger proportion both of casein and saline matter. Besides the ordinary fat globules, it appears to contain a number of larger, irregular, granular particles, which gradually disappear with the subsidence of the milk fever. Milk long detained in the breast becomes watery and impoverished, so that that which flows last during the operation of suckling is the richest in solid matter. If menstruation occur during lactation the milk becomes more serous, and frequently disagrees with the infant. Violent mental emotions of various descriptions appear to modify the secretion in a manner more irregular and less perfectly understood. From some experiments made by our author, he finds that the milk from brunettes is in general richer than that furnished by women of fair complexion. Various compounds pass from the system into the milk. "Common salt, sulphate of soda, iodide of potassium, oxide of iron, trisnitrate of bismuth, passed into the milk of asses." (p. 638.) Mercury probably does the same, judging from the effect of the medicine upon syphilitic infants when administered to their nurses.

The tenth chapter is devoted to the Locomotive Apparatus.

*Bone.* The composition of the bones varies with the different parts of the body from which they are taken. The proportion of animal matters to the earthy may be stated in round numbers as 2 to 3. Their constituents in the human subject are gelatin, (or a tissue which when boiled furnishes this principle,) phosphate and carbonate of lime, phosphates of magnesia, soda, and chloride of sodium. Fluoride of calcium has been mentioned among the constituents of the bones, but Dr. Rees has shown that it admits of considerable doubt whether it really does exist in them. The bones have been subjected to analysis in various diseases. In caries and necrosis no chemical alteration has been detected. In rickets and mollities ossium the proportion of earthy matters has been found so much reduced as to bear to the animal matters the ratio of only 1 to 3, and the proportion of phosphate of lime diminishes in somewhat greater proportion than that of the other saline components. Permanent callus differs little in composition from ordinary bone; when first deposited, the organic compounds predominate, and the phosphate of lime bears a smaller ratio to the other salts than in healthy bone. The *teeth* are more compact than ordinary bone, and contain about 3-10ths of organic matter. They are occasionally encrusted with a calcareous deposit, known as the tartar of the teeth, consisting of about 80 per cent. of phosphates of lime, and the remainder of mucus and the constituents of the saliva.

Little of interest is known respecting the changes which the *muscular system* undergoes. Fibrin forms the essential ingredient in its composition, but in certain degenerations of the tissue we find the true muscular fibre replaced by a peculiar saponifiable fat, the nature of which has not been accurately ascertained. In a few instances the texture has been found ossified and composed of the same ingredients as healthy bone. The aponeurotic, tendinous, and ligamentous tissues contain, besides albumen, a considerable proportion of matter convertible by boiling into gelatin; but this principle does not appear to exist ready formed in them.

The concluding chapter of the work is devoted to Morbid Products.

*False membrane.* The principal ingredients in this are albumen, in its coagulated and uncoagulated form, and fibrin. In one instance, analysed by L'Heritier, cholesterin was found to be present.

*Pus.* This product, when healthy, has a specific gravity of 1031 to 1033. It is generally neutral to litmus; by exposure to air, it soon undergoes decomposition. It varies greatly in appearance with the nature of the surface from which it is secreted. Nothing definite respecting the chemical differences presented by these varieties is known. Pus has been found by analysis to contain albumen, lactates, chloride of sodium, phosphate of lime, with a small quantity of iron. Respecting its microscopic appearance, we have nothing to add to the quotation already made when speaking of its occurrence in the urine. Treated with ether, pus gives up a certain proportion of fatty matter, by which Rayer proposes to distinguish it from mucus; but this is a vague and uncertain character, as fat may find its way into the different secretions from other sources than the formation of pus, and Dr. Bird found only 0·5 per cent. of fat in pus which he analysed from a psoas abscess. Our author seems to favour the exploded doctrine of a resolution of certain parts of the solids into pus, and we shall therefore not be surprised to find his ideas on its formation rather hazy and ill developed.

*Tubercles.* After successfully refuting, both on chemical and physical grounds, the hypothesis of Lallemand and Cruveilhier, that tubercles arise from concrete pus, he furnishes us with the following excellent specimen of the art of wrapping up ignorance in the garb of science, and passing it off upon the unwary under the guise of sterling knowledge:

"It is with tuberculous matter as with pus. It is a heterogeneous product, capable, in my opinion, of developing itself under the influence of a variety of causes whenever the tuberculous diathesis exists. Like pus it may proceed from metamorphosed blood; like it also it may consist in the transformation of a normal or abnormal tissue.

"In all these cases the formation of tuberculous matter depends upon the inability of the tissue to assimilate, that is to say, to convert into its normal substance the materials brought for its nutrition; whether because the materials resist the transformation, or because the assimilating power of the tissue cannot exert itself with sufficient energy to master the material to be assimilated. The principal cause depends either upon the plastic activity in general being altered from its normal conditions, upon the formation of the blood remaining incomplete, or else upon the degeneration of this fluid." (p. 680.)

Vogel gives the following account of the microscopic examination of tubercular matter. After directing a thin slice to be placed under the object glass, he says—

"We see now in the proper substance of the tubercle absolutely no blood-vessels, and convince ourselves by repeated examinations of such fresh sections, that the substance of the tubercle itself not only contains no blood-vessels, but that by its deposition in the pulmonary tissue the original vessels of the lungs have been compressed and obstructed. We discover, on the other hand, the serpentine fibrous bundles of the substance of the lungs forming meshes, and still entirely unaltered between the masses of tubercle. If, concealed by masses of tubercle they do not at once appear evident, they will do so on adding ammonia, which renders the substance of the tubercle transparent. If we examine the substance of the tubercle more accurately, by washing with water, rubbing it down, tearing it with needles, and so forth, and seek to resolve a fine section into its morphological elements, we shall convince ourselves that it consists entirely of an aggregation of the tubercle-cells already mentioned, without any connecting medium." (Vogel, p. 458.)

These cells are thus described:

"In different cases of tubercular disease, and even commonly upon the same spot of the same tubercle, the cells exhibit great differences. Sometimes they are small, only from 1-200 to 1-400 of a line in diameter, for the most part roundish, with pale outlines; their nuclei are proportionately large, from 1-300 to 1-500 of a line in diameter, and are commonly roundish, rather dark, with or without nucleoli; they fill up almost the whole cell, which contains no solid particles beside the nucleus; in other cases the cells are larger, 1-80 to 1-200 of a line in diameter, sometimes roundish, sometimes oval, at others of irregular form, elongated or drawn out into a tail; their nuclei are smaller in proportion to the size of the cell, and occupy only a third or fourth part of its capacity. These cells, besides their nuclei, often contain small globules of fat, in greater or less number. . . . . In other cases the cells contain granules of a black pigment.

"We find that the tissue of the lungs infiltrated with tuberculous matter, passes quite gradually into the healthy tissue, and in the former we never find anything resembling a membrane inclosing the tubercle. The surrounding pulmonary tissue appears sometimes normal, sometimes filled with nucleated cells, a consequence of the inflammation excited by the pressure of the tuberculous mass upon the neighbouring parts." (Ib. p. 457-9.)

The principal chemical constituent of tuberculous, like that of all malignant deposits, is albumen; and in addition, gelatin, fibrin, fatty matters, phosphate and carbonate of lime are enumerated. In hardened tubercles, L'Heritier found 5 to 9 per cent. of animal matters, and from 91 to 95 of a mixture of carbonate and phosphate of lime.

In *colloid* matter gelatin is said to preponderate.

The *encephaloid*, or medullary tumour, appears to contain, besides albumen and fibrin, gelatin, phosphorized fat, and occasionally cholesterin, besides saline matters. The deposit in *melanosis* consists principally of albuminous and fibrous matter, with a variable proportion of fat, coloured with an organic substance, soluble in dilute sulphuric acid, and resembling in everything but its colour the hematosin of the blood. Phosphate of lime and iron are found among the saline ingredients. Except in the absence of the colouring matter, and in the variation of the proportions of its constituents, which here, as well as in melanosis, vary greatly, the matter of *scirrhus* differs little in composition from that of the last-named disease.

*Calculous concretions.* These occur in various parts of the body, and as they are derived from the deposition of sparingly soluble compounds contained in the different fluids, they naturally vary with the situation in which they occur, and the composition of the secretions from which they are separated. Under the prepuce, concretions have been found, consisting of phosphate of lime, mucus, and fatty matter. From the tonsils, larynx, nose, salivary, lacrymal, and pancreatic ducts, and bronchi, calculi have been taken, consisting of carbonates and phosphates of lime and magnesia, mucus, and fatty matter. The proportions of these vary greatly, and the magnesia is always small in quantity. Arthritic calculi, besides phosphate of lime, generally furnish a considerable proportion of the urates of soda and lime.

*Intestinal calculi* our author divides into three classes, according to their origin:

"1st. Those which originate in nuclei formed either in the alimentary canal or biliary apparatus, but which are covered with saline particles or animal matters during their passage through the intestines: 2dly, those which originate in nuclei composed by foreign bodies, such as seeds, stones of fruit, the husks of fruits, fragments of bones, &c., covered with crystalline particles which would not have become aggregated but for the presence of these foreign bodies; and 3dly, those which are entirely formed in the digestive tube which are homogenous, and present no distinct nuclei. It is not very rare to find in the alimentary canal, and more particularly in the large intestine, concretions entirely different from those of which I have just spoken. Some are concreted masses ordinarily derived from three sources: 1st, A morbid state of the secretions poured into the digestive tube or secreted upon its surface; 2dly, an alteration of the fecal matters which occasionally become charged with earthy particles during their retention in the cæcum and in the large intestine; and 3dly, the introduction into the stomach of substances not susceptible of digestion. Others are of a fatty or adipocirous nature." (p. 695-7.)

*Biliary calculi.* These also consist of three principal varieties:—the *lamellated*, which are hard and but slightly inflammable; the *radiated*, or striated, which bear a crystalline semi-transparent appearance, and often a granular surface,—they consist of cholesterin nearly pure; and the *corticated,* consisting of a lamellated nucleus of cholesterin, enveloped by an intermediate layer, over which is a crust or bark of infusible matter. Besides cholesterin, which is the principal ingredient of most biliary calculi, the following substances have been met with: The yellow and green colouring matter of bile, picromel (or bilin), mucus, albumen (?), carbonaceous matter, carbonates of lime and soda, phosphates of lime and magnesia, and oxide of iron.

Our author concludes his treatise with a brief summary of the most important facts relative to *urinary calculi.* A neat sketch of the methods of detecting the presence of the different ingredients is given; and a few pages are devoted to the chemical treatment of calculous disorders, in which considerable stress is laid upon the method of treatment by alkalies. The expectations of success held out are considerably more sanguine than we are warranted in forming from experience.

In conclusion, we may safely recommend the treatise of M. L'Heritier, as a valuable aid in the study of chemical pathology. Although he is apt to be prolix in unimportant matters, and to subdivide his subjects to excess, his work will be found a judicious compilation of most that has been done upon the subject, and we have pleasure in adding that the writer appears to have studied with care the researches of foreigners as well as those of his own countrymen, though he has not always been sufficiently careful in stating the source whence his information is derived: he has at the same time added many valuable analyses and observations of his own. The work is, however, deficient in the terseness, close reasoning, and laborious accumulation of facts which distinguish that of Lehmann; and in the novelty and originality of investigation which mark the treatise of Vogel.

From the review we have just taken of one of the most recent treatises on pathological chemistry, we see how vague and incomplete our knowledge is upon almost every part of this important branch of science. We are as yet but just entering upon its study. Great difficulties in its prosecution have still to be overcome. These arise in part from our ignorance, in part from the very nature of the subject itself. In no department of inquiry is a more scrupulous attention to literal accuracy necessary, inasmuch as the observations and analyses being made upon peculiar and individual cases are not susceptible of the severe verification and close scrutiny by other observers, which researches in many other departments of science admit, and from which we derive such a feeling of exactness and certainty in the deductions obtained. Speculations of high interest have been thrown out; the field of investigation is new; many ardent inquirers have entered upon it, eager to obtain a reputation where labour and accuracy are alone needed to ensure a creditable station. The labour is willingly given, but the accuracy which alone can render that labour valuable, has too often been forgotten or neglected, either from the inadequacy of those who have undertaken the task to execute it satisfactorily or, what is still less excusable,—from indolence or over-hastiness, and consequent omission of the needful precautions. Great, however—even in this early stage, and notwithstanding our comparative ignorance—are the benefits that have accrued to the science of medicine from the prosecution of Pathological Chemistry; and proportionately great, therefore, is the encouragement we may derive for its further cultivation; as it is impossible to doubt that increased knowledge will add to the precision and fitness of our remedial measures, as well as to the accuracy of that upon which all treatment must be based, our diagnosis of disease.

The researches of the present day seem all converging towards one point, viz. the mutual convertibility of the various species of force; rendering it probable that the amount of force existing on our planet is as definite as that of matter itself. The leading idea in Liebig's treatise is the dependence of all manifestations of vitality upon a definite and corresponding amount of chemical force; an idea at present wrought out, it is true, but imperfectly, and often supported by this ingenious philosopher rather by sweeping analogies and bold speculation than by sober experiment and careful observation: but the general correspondence of facts with the theory recommends it strongly to our notice, and the simplicity and beauty of the hypothesis almost irresistibly leads the judgment captive. The close mutual dependence of the forces of electricity and magnetism is now established beyond the shadow of a doubt; a connexion so intimate that a correct estimate of the amount of the one forms the most accurate measure of the quantity of the other,—a connexion so intimate that from the same source we may at pleasure obtain either, and when obtained, we may, by a slight modification of our arrangements, convert the one into the other, as is strikingly exemplified in that invaluable instrument the galvanometer.

Not less closely related to electricity and magnetism is the equally subtle and important agency of heat. The researches of Melloni, who, in the beautiful thermomultiplier devised by Nobili and himself, applied Seebeck's discovery of thermo-electricity to the investigation of the laws of radiant heat, have shown that the deviation of the magnetic needle, produced by an electric current excited by the rays of heat falling upon the exposed surface of the multiplier, may be employed as an accurate measure of the quantity of heat which falls upon the instrument at any given moment; and we are all familiar with the converse of the fact in the intense evolution of heat produced during the passage of electricity through imperfect conductors, a property of which the chemist avails himself to fuse the most refractory substances in nature, and which may, under due regulation, as in Snow Harris's experiments, be employed as an exact measure of the force in circulation. Faraday has taught us that we may convert a given amount of electric action into a chemical action equally definite in quantity, and, *vice versâ*, that chemical action may be made to produce a corresponding and equivalent development of electricity, and if of electricity, an equivalent amount also of magnetism and of heat. Indeed, the definite quantity of heat, disengaged by chemical action, has been ascertained by direct experiment, independent of the electricity set free, and to chemical action we ordinarily have recourse for the production of artificial heat.

Here are four of the subtlest agents of the material universe related by the closest ties. The measure of the one becomes a measure of each of the others, and doubtless, could we make the suitable arrangements, we should always find one force associated with a corresponding amount of each of the others. They appear not to be independent forces, each having a separate existence, but merely different manifestations of one and the same invisible and imponderable agent.

Further, mechanical force may be converted into magnetic, and magnetic again into chemical. *E. g.* In a patent recently taken out, gravitation, by the falling of a weight, or elasticity, by the uncoiling of a spring, causes a piece of soft iron, properly surrounded by wire, to revolve rapidly in front of the poles of a horse-shoe magnet; an electric current is induced in the wire, and this current is employed for precipitating metallic copper from its solutions, and a voltatype impression is thus obtained by the conversion of mechanical into chemical force. Chemical force is again converted into mechanical in every steam-engine at work in our land; for every cwt. of oxygen that combines with the coal or coke of its furnace, an equivalent amount of water is, or may be, converted into steam, and the elasticity of this steam is then made to perform, at the will of him that raised it, any species of mechanical work to which it shall please him to apply it. Transformations of force analogous to these seem to keep the universe in motion ; its varied phenomena resolve themselves into the transfer and transformation of forces. In all human inventions, however, for the transformation of force, power is lost ; we cannot employ the whole of that which is set free ; our contrivances are imperfect, and our methods of economising and measuring the forces with which we deal are incomplete and inaccurate. Not so with the contrivances of the Divine artificer : He knows exactly what arrangements are required to employ the whole power He sets in motion : and may we not, without extravagance, suppose that the varied phenomena which constitute the sum total of vitality may result from the simultaneous employment, by appropriate channels, of *all* the forces developed at the instant that any *one* of those forces (as we are in the habit of considering them) is developed ? Is it impossible that by the inscrutably delicate arrangements of organization scope may be given to the manifestation of another modification of force which we term vitality ? Or is it too much to imagine that life is maintained not by chemical agency alone, but by the union of all the forces set in motion whenever chemical action, as being the most convenient in its adaptation to our frame, is excited ?

Leaving speculation, however, let us descend to the realities of fact, enjoining upon ourselves a double caution lest, warped by preconceived theories, our judgment should err, and we should, as the most ingenuous minds often unwittingly feel tempted to do, bend or gloss over our facts till they accommodate themselves to our own crude hypotheses as to what *must* be, instead of contenting ourselves with the discovery of things as they actually exist.

